# Association Between Lipoprotein(a) and Peri-procedural Myocardial Infarction in Patients With Diabetes Mellitus Who Underwent Percutaneous Coronary Intervention

**DOI:** 10.3389/fendo.2020.603922

**Published:** 2021-02-03

**Authors:** Yupeng Liu, Wenyao Wang, Jingjing Song, Kuo Zhang, Bo Xu, Ping Li, Chunli Shao, Min Yang, Jing Chen, Yi-Da Tang

**Affiliations:** ^1^ Department of Cardiology, State Key Laboratory of Cardiovascular Disease, Fuwai Hospital, National Center for Cardiovascular Diseases, Chinese Academy of Medical Sciences and Peking Union Medical College, Beijing, China; ^2^ Department of Cardiology, Peking University Third Hospital, Beijing, China; ^3^ Graduate School of Peking Union Medical College, Chinese Academy of Medical Sciences and Peking Union Medical College, Beijing, China; ^4^ Department of Cardiac Catheterization Laboratory, State Key Laboratory of Cardiovascular Disease, Fuwai Hospital, National Center for Cardiovascular Diseases, Chinese Academy of Medical Sciences and Peking Union Medical College, Beijing, China

**Keywords:** lipoprotein(a), peri-procedural myocardial infarction, diabetes mellitus, percutaneous coronary intervention, coronary artery disease

## Abstract

**Background:**

High lipoprotein(a) (Lp[a]) levels are associated with increased risks of cardiovascular events in Percutaneous Coronary Intervention (PCI) patients with diabetes mellitus (DM). Peri-procedural myocardial infarction (PMI) occurs commonly during the PCI, whereas the relationship between Lp(a) and PMI remains unclear. Our study aimed to evaluate the association between Lp(a) value and the incidence of PMI in a larger-scale diabetic cohort undergoing PCI throughout 2013.

**Methods:**

A total of 2,190 consecutive patients with DM were divided into two groups according to the median Lp(a) level of 175 mg/L: Low Lp(a) group (N = 1095) and high Lp(a) group (N = 1095). PMI was defined based on the 2018 universal definition of myocardial infarction.

**Results:**

Patients with high Lp(a) levels exhibited higher rates of PMI compared to those with low Lp(a) levels (2.3% versus 0.8%, P = 0.006). The multivariable logistic analysis showed that PMI was independently predicted by Lp(a) as a dichotomous variable (OR 2.64, 95%CI 1.22–5.70) and as a continuous variable (OR 1.57, 95% CI 1.12–2.20). However, further investigation found that this association was only maintained in men, whose Lp(a) levels were significantly associated with the frequency of PMI, both as a dichotomous variable (OR 3.66, 95%CI 1.34–10.01) and as a continuous variable (OR 1.81, 95%CI 1.18–2.78). Lp(a) wasn’t a risk factor of PMI in women.

**Conclusions:**

High Lp(a) levels had forceful correlations with the increased frequency of PMI in male diabetic patients undergoing PCI. Lp(a) might act as a marker of risk stratification and a therapeutic target to reduce PCI-related ischemic events.

## Introduction

It is estimated that the number of patients with diabetes mellitus (DM) has doubled in the past two decades (1). DM has become one of the most common metabolic disorders in contemporary PCI practice. DM exerts the pro-thrombotic and pro-inflammatory effects in the pathogenesis of atherosclerosis and increases the risk of cardiovascular consequences (2). It’s noteworthy to identify the risk factors that predict cardiac events in diabetic patients undergoing PCI.

Peri-procedural myocardial infarction (PMI) is a procedure-related ischemic cardiac event following coronary stenting (3). There is a growing body of evidence showing the association between PMI and worse outcomes after PCI over short- and long-term periods (4–6). Previous research proposed that the distal and side-branch thromboembolism, which resulted from lipid-rich plaques rupture, gave rise to the occurrence of PMI (7). Moreover, blood lipid profiles, including high-density lipoprotein (HDL), low-density lipoprotein (LDL), and fatty acid were demonstrated to have correlations with PCI-related myocardial injury (8–10).

Lipoprotein(a) (Lp[a]) is a LDL-like lipid with additional apolipoprotein(a) attached to apolipoprotein B (11). Epidemiological studies identified Lp(a) as an atherothrombotic risk factor, and prior studies revealed the relationship between the elevation of Lp(a) and the myocardial ischemic events (12, 13). But much less is known about the impact of elevated Lp(a) on the peri-procedural coronary events among patients with DM.

Therefore, our study aimed to investigate whether high Lp(a) status was associated with an increased risk of PMI, defined by the latest 2018 fourth universal definition of myocardial infarction (UDMI) (14), among a large-scale diabetic cohort undergoing PCI. Univariable and multivariable logistic regression models and receiver-operating curve (ROC) analysis were used to investigate the predictive value of Lp(a).

## Material and Methods

### Study Population

The study enrolled consecutive patients with DM undergoing PCI at Fuwai Hospital (Beijing, China) between January 2013 and December 2013. Patients without available Lp(a) and cardiac biomarker data were excluded. Cardiac markers were generally measured in patients undergoing PCI, while the measurement of Lp(a) wasn’t routinely performed. Therefore, we compared the differences between patients with and without available Lp(a) data. As shown in **Supplementary Table S1**, most baseline characteristics and procedural features were evenly distributed between patients without available Lp(a) data and with available Lp(a) data. All patients had written informed consent. The study complied with the principles of Helsinki and was approved by the hospital review board. A total of 2,190 subjects were eligible for the present analysis. According to the median Lp(a) level of 175 mg/L, patients were divided into two parts: Low-Lp(a) group (N = 1,095) and High-Lp(a) group (N = 1,095). Baseline clinical and procedural data were extracted from medical records. Venous blood was collected from all patients after fasting overnight. Biochemical analyses were performed in the biochemistry laboratory of Fuwai Hospital. Lp(a) concentrations were measured by immunoturbidimetry (Lasay Lp(a) auto, Shima laboratories, Japan) method following the manufacturer’s instructions.

### Procedure

The PCI strategies and stents were determined by treating cardiologists according to patients’ characteristics. A loading dose of 300 mg aspirin and 300 mg clopidogrel was prescribed for the patients before the procedure. After the procedure, all patients received at least 1 year of clopidogrel (75 mg/day) and lifelong aspirin (100mg/day). The Synergy between Percutaneous Coronary Intervention (PCI) with TAXUS and Cardiac Surgery (SYNTAX) score (http://www.syntaxscore.com) was calculated by treating cardiologist at Fuwai Hospital. PCI with a SYNTAX score of more than 22 were considered a high-risk PCI procedure (15). Levels of cardiac biomarkers were assayed before and after procedures. The diagnostic definition of PMI was based on the 2018 UDMI. PMI was defined as a rise of more than five times the 99th percentile upper reference limit (URL) of cardiac biomarker levels when patients showing normal baseline levels or an increase over 20% cardiac biomarker levels when patients showing elevated baseline levels but stable or declining. Additional evidence of ischemic ECG findings, imaging changes, and angiographic complications was required for the diagnosis.

### Statistical Methods

Continuous characteristics were presented as mean ± SD and comparisons were made with Student’s t‐test. Categorical variables were described as frequencies with percentages and evaluated with the chi‐square test or Fisher’s exact test as appropriate. Univariate and multivariate logistic regression evaluations were performed to identify the independent predictors for PMI. Lp(a) was presented as a categorical variable [high Lp(a)] and a continuous variable (natural logarithm) in the analysis. The receiver-operating curve (ROC) was used to assess the prediction for PMI. Kaplan-Meier survival estimate was used to evaluate time-to-event data. All P values were two-sided, and a P-value <0.05 was considered statistically significant. Statistical analyses were performed using SPSS version 22.0 (SPSS, Inc., Chicago, IL).

## Results

### Patients Characteristics

Patients were stratified into high Lp(a) group and low Lp(a) group and major clinical and angiographic variables of the study population are shown in **Tables 1** and **2**. 75.7% of patients were male and their mean age was 59 years old.

**Table 1:** Baseline characteristics of study patients.

	Low Lp(a) (N = 1095)	High Lp(a) (N = 1095)	P Value
Age	59.0 ± 9.7	59.6 ± 9.8	0.118
Male	861 (78.6)	796 (72.7)	<0.001
BMI	26.4 ± 3.1	26.1 ± 3.2	0.030
Current smoking	632 (57.7)	580 (53.0)	0.025
Hypertension	771 (70.4)	766 (70.0)	0.815
Hyperlipidemia	818 (74.7)	815 (74.4)	0.883
Previous CABG surgery	45 (4.1)	60 (5.5)	0.134
Previous PCI	284 (25.9)	186 (26.1)	0.922
Previous MI	343 (47.2)	383 (52.8)	0.069
Peripheral vascular disease	50 (4.6)	49 (4.5)	0.918
Previous cerebrovascular disease	152 (13.9)	151 (13.8)	0.951
Total cholesterol, mmol/L	4.1 ± 1.1	4.2 ± 1.1	0.003
Lipoprotein(a), mg/L	76.8 ± 46.7	486.4 ± 271.7	<0.001
HDL, mmol/L	1.0 ± 0.3	1.0 ± 0.3	0.008
LDL, mmol/L	2.3 ± 0.9	2.5 ± 1.0	<0.001
hs-CRP, mg/L	2.9 ± 3.4	3.6 ± 4.0	<0.001
HbA1c	7.7 ± 1.4	7.9 ± 1.5	0.008
Creatinine, μmoI/L	75.2 ± 16.7	75.1 ± 16.9	0.878
LVEF	62.9 ± 7.5	62.4 ± 7.5	0.169

^a^Variables are shown as median ± SD or n (%).

Lp(a), lipoprotein(a); PCI, percutaneous coronary intervention; MI, myocardial infarction; CABG, coronary artery bypass graft; CAD, coronary artery disease; BMI, body mass index; LVEF, left ventricular ejection function; HDL, high-density lipoprotein; LDL, low-density lipoprotein; hs-CRP, high sensitivity C-reactive protein; HbA1c, haemoglobin A1c.

**Table 2:** Procedural characteristics of study patients.

	Low Lp(a) (N = 1095)	High Lp(a) (N = 1095)	P Value
Multivessel disease	893 (81.6)	921 (84.1)	0.113
Left main disease	37 (3.4)	48 (4.4)	0.224
Bifurcation lesion	427 (39.0)	473 (43.2)	0.046
Total occlusion	170 (15.5)	189 (17.3)	0.273
Target vessel diameter, mm	3.1 ± 1.4	3.1 ± 0.5	0.210
Percent stenosis	88.4 ± 8.3	89.2 ± 8.1	0.038
Total treated lesion length, mm	29.7 ± 18.7	30.4 ± 18.7	0.382
Moderate to severe calcification	201 (18.4)	188 (17.2)	0.467
Moderate to severe angulation	94 (8.6)	99 (9.0)	0.706
SYNTAX score	11.9 ± 8.2	12.5 ± 8.4	0.069
*Baseline TIMI flow grade*			
TIMI 0	174 (15.9)	198 (18.1)	0.172
TIMI 1	47 (4.3)	43 (3.9)	0.667
TIMI 2	97 (8.9)	131 (12.0)	0.017
TIMI 3	777 (71.0)	723 (66.0)	0.013
*Stent type*			
Bare-metal stents	7 (0.6)	5 (0.5)	0.563
Drug-eluting stents	1,026 (93.7)	1,037 (94.7)	0.315
Number of stents	1.8 ± 1.2	1.9 ± 1.2	0.043
Procedural duration, min	34.9 ± 31.6	37.2 ± 31.2	0.081

^a^Variables are shown as median ± SD or n (%).

Lp(a), lipoprotein(a); TIMI, thrombolysis in myocardial infarction.

High Lp(a) group were more frequently female, as well as showing more complicated procedural features, compared with low Lp(a) group. As expected, significant worse metabolic profiles exhibited in the high Lp(a) group, which were composed of high LDL levels, and high Haemoglobin A1c (HbA1c) levels.

### Lp(a) and PMI

**Figure 1:**
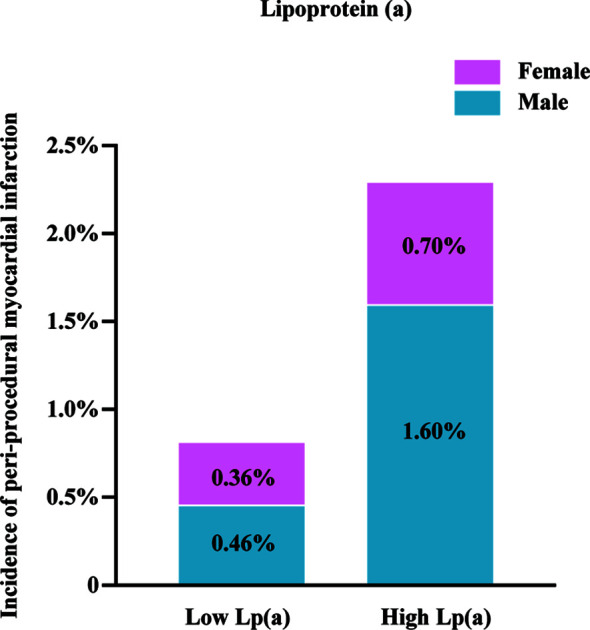
Incidence of peri-procedural myocardial infarction. Lp(a), lipoprotein(a); high Lp(a), Lp(a) > 175 mg/L.

**Figure 1A** demonstrates the proportion of patients with PMI according to the Lp(a) levels. Patients with high Lp(a) levels exhibited an increased occurrence of PMI than those with low Lp(a) levels (2.30% versus 0.80%, P=0.006). In male patients, the incidence of PMI was considerably higher in the high Lp(a) group than the low Lp(a) group (1.60% versus 0.46%, P=0.006). In female patients, the incidences of PMI between the two groups were not significantly different (0.76% versus 0.36%, P=0.456). **Table 3** provides the univariable logistic regression analysis for the prediction of PMI, which summarizes that Lp(a), as a continuous variable [log-transformed Lp(a)] (OR 1.64, 95%CI 1.17–2.30, P = 0.004) or a dichotomous variable [high Lp(a)] (OR 2.82, 95% CI 1.31–6.07, P = 0.008), was significantly associated with the increased frequency of PMI. However, after separately exploring the association between Lp(a) and PMI in males or females, we found no significant relationship of Lp(a) to PMI in females. **Table 4** shows the multivariable models for the prediction of PMI. High Lp(a) levels were independently associated with increased incidence of PMI than low Lp(a) level [adjusted OR 2.64, 95% CI 1.22–5.70, P = 0.014]. Besides, the relationship between the log-transformed Lp(a) and elevated incidence of PMI was also significant (adjusted OR 1.57, 95%CI 1.12–2.20, P = 0.008). Consistently, after adjustment, Lp(a) was still the risk factor of PMI in men as a dichotomous variable (adjusted OR 3.66, 95%CI 1.34–10.01, P=0.011) or a continuous variable (adjusted OR1.81, 95%CI 1.18–2.78, P=0.007), whereas Lp(a) did not predict PMI events in women.

**Table 3:** Univariate logistic regression analysis for peri-procedural myocardial infarction.

	Total population		Male		Female	
	Odds ratio (95% CI)	P Value	Odds ratio (95% CI)	P Value	Odds ratio (95% CI)	P Value
Age	2.20 (1.07–4.53)	0.033	1.05 (1.01–1.10)	0.021	0.995 (0.93–1.07)	0.885
Male	0.58 (0.29–1.19)	0.138	Not applicable		Not applicable	
BMI	1.09 (0.99–1.20)	0.086	1.09 (0.97–1.23)	0.139	1.10 (0.93–1.30)	0.283
Hypertension	1.18 (0.55–2.55)	0.668	1.03 (0.42–2.54)	0.953	1.40 (0.30–6.48)	0.667
Current smoking	0.56 (0.28–1.11)	0.098	0.63 (0.27–1.47)	0.283	0.74 (0.09–5.84)	0.775
LVEF	0.97 (0.94–1.02)	0.621	0.97 (0.92–1.01)	0.153	0.99 (0.91–1.07)	0.760
Previous MI	0.92 (0.47–1.99)	0.921	0.96 (0.40–2.31)	0.932	1.31 (0.35–4.91)	0.693
High LDL	1.13 (0.55–2.33)	0.884	1.05 (0.44–2.51)	0.918	1.16 (0.31–4.33)	0.829
LDL, mmol/L	1.06 (0.73–1.53)	0.754	0.99 (0.60–1.62)	0.963	1.07 (0.61–1.88)	0.814
HbA1c <7%	0.60 (0.27–1.33)	0.204	0.68 (0.26–1.76)	0.428	0.50 (0.11–2.31)	0.374
HbA1c	1.17 (0.94–1.44)	0.163	1.17 (0.89–1.52)	0.263	1.13 (0.78–1.63)	0.527
Low HDL	1.09 (0.55–2.14)	0.814	1.14 (0.48–2.73)	0.769	0.84 (0.25–2.81)	0.772
HDL, mmol/L	0.76 (0.21–2.72)	0.676	0.47 (0.08–2.81)	0.405	0.81 (0.11–5.95)	0.833
High Lp(a)	2.82 (1.31–6.07)	0.008	3.74 (1.37–10.17)	0.010	1.58 (0.47–5.32)	0.459
Ln-Lp(a)	1.64 (1.17–2.30)	0.004	1.84 (1.20–2.84)	0.006	1.26 (0.74–2.15)	0.395
SYNTAX score >22	2.97 (1.41–6.29)	0.004	1.07 (0.31–3.63)	0.919	12.11 (3.71–39.47)	<0.001
SYNTAX score	1.05 (1.01–1.08)	0.017	1.01 (0.96–1.06)	0.677	1.12 (1.05–1.19)	0.001
Total lesion length	1.01 (0.99–1.02)	0.359	1.004 (0.98–1.03)	0.709	1.02 (0.99–1.04)	0.254
Number of stents	0.97 (0.72–1.30)	0.842	0.94 (0.65–1.37)	0.747	1.03 (0.64–1.67)	0.899

^a^95%CI, 95% confidence interval; Lp(a), lipoprotein(a); LVEF, left ventricular ejection function; MI, myocardial infarction; LDL, low-density lipoprotein cholesterol; HDL, high-density lipoprotein cholesterol; HbA1c, Haemoglobin A1c.

High Lp(a), Lp(a) > 175 mg/L; high LDL, LDL > 2 mmol/L; low HDL, HDL < 1 mmol/L; ln-Lp(a), natural log Lp(a).

**Table 4:** Multivariate logistic regression analysis for peri-procedural myocardial infarction.

	Total population		Male		Female	
	Odds ratio (95%CI)	P Value	Odds ratio (95%CI)	P Value	Odds ratio (95%CI)	P Value
Lp(a) as categorical variable						
Age	1.03 (0.996–1.07)	0.079	1.05 (1.01–1.10)	0.027	0.98 (0.92–1.05)	0.630
Male	0.74 (0.35–1.57)	0.435	Not applicable		Not applicable	
High Lp(a)	2.64 (1.22–5.70)	0.014	3.66 (1.34–10.01)	0.011	1.28 (0.37–4.47)	0.695
Previous MI	0.95 (0.454–1.99)	0.893	0.89 (0.37–2.15)	0.798	1.001 (0.25–4.05)	0.999
SYNTAX score >22	2.80 (1.31–5.95)	0.008	0.99 (0.29–3.41)	0.993	12.19 (3.67–40.48)	<0.001
Lp(a) as continuous variable						
Age	1.03 (0.995–1.07)	0.094	1.05 (1.004–1.10)	0.031	0.98 (0.92–1.05)	0.635
Male	0.78 (0.37–1.65)	0.518	Not applicable		Not applicable	
Ln-Lp(a)	1.57 (1.12–2.20)	0.008	1.81 (1.18–2.78)	0.007	1.17 (0.66–2.07)	0.601
Previous MI	0.93 (0.44–1.94)	0.841	0.87 (0.36–2.10)	0.754	0.97 (0.24–3.97)	0.967
SYNTAX score >22	2.82 (1.32–5.998)	0.007	0.99 (0.29–3.40)	0.989	12.10 (3.65–40.17)	<0.001

^a^95%CI, 95% confidence interval; Lp(a), lipoprotein(a).

High Lp(a), Lp(a) > 175 mg/L; ln-Lp(a), natural log Lp(a).

**Figure 2:**
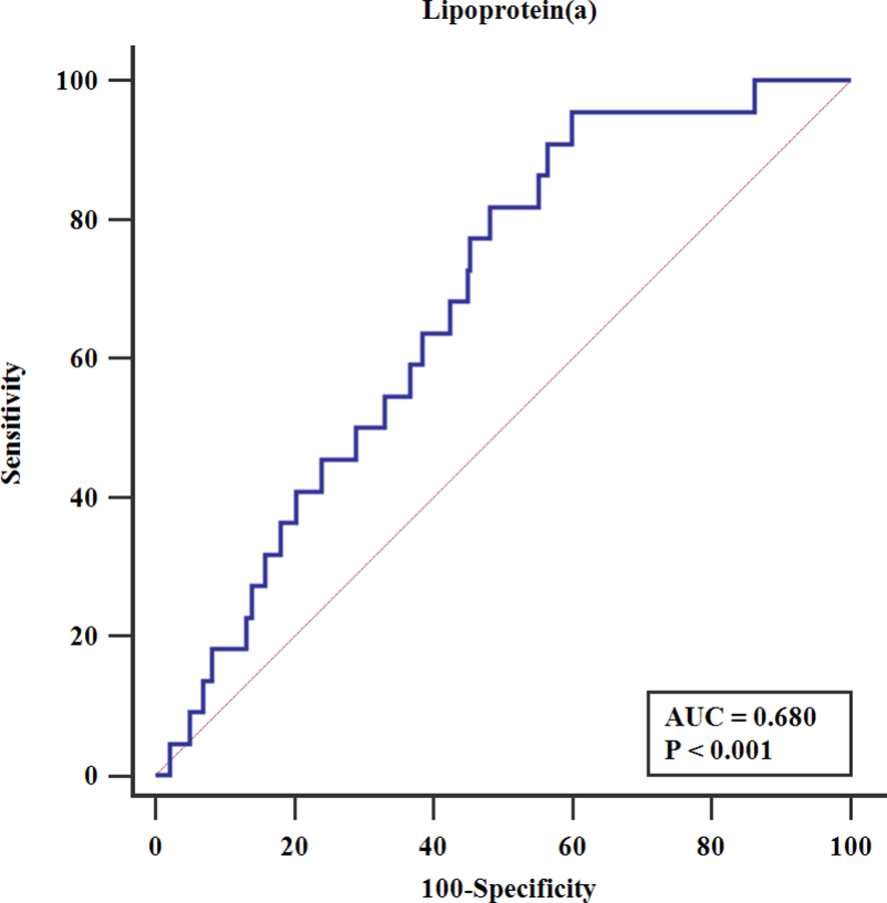
Receiver-operating characteristics analysis. AUC, the area under the receiver-operating curve.

To further provide an estimate that might be compared against in the future, we conducted ROC analysis in male diabetic patients. **Figure 2** shows that the area under the receiver-operating curve (AUC) was 0.680 (95% CI 0.657–0.703, P <0.001) for Lp(a) to predict PMI. The optimal cut-off value was 112.4 mg/L (AUC=0.678, sensitivity 95.5%, specificity 40.1%).

## Discussion

### Major Findings

The major findings of our research were high Lp(a) was independently associated with the occurrence of PMI and a predictor for PMI in diabetic patients undergoing PCI, but further investigation found the association was only significant in male, and Lp(a) wasn’t a risk factor of PMI for female. Our analysis indicated that high Lp(a) might be a convenient screening marker for the assessment of PMI, and reducing Lp(a) concentrations might be one possible way to improve the outcome of PCI patients with DM.

### The Clinical Implication of Lp(a) and PMI in Patients With DM

It is reported that diabetic patients had a two-fold higher risk for cardiovascular diseases than non-diabetic patients (16). However, previous studies demonstrated that intensive lowering of glucose levels didn’t reduce the risk of cardiac events (17). Thus, it is of great importance to find out the risk factor for diabetic patients to prevent adverse cardiovascular consequences.

**Figure 3:**
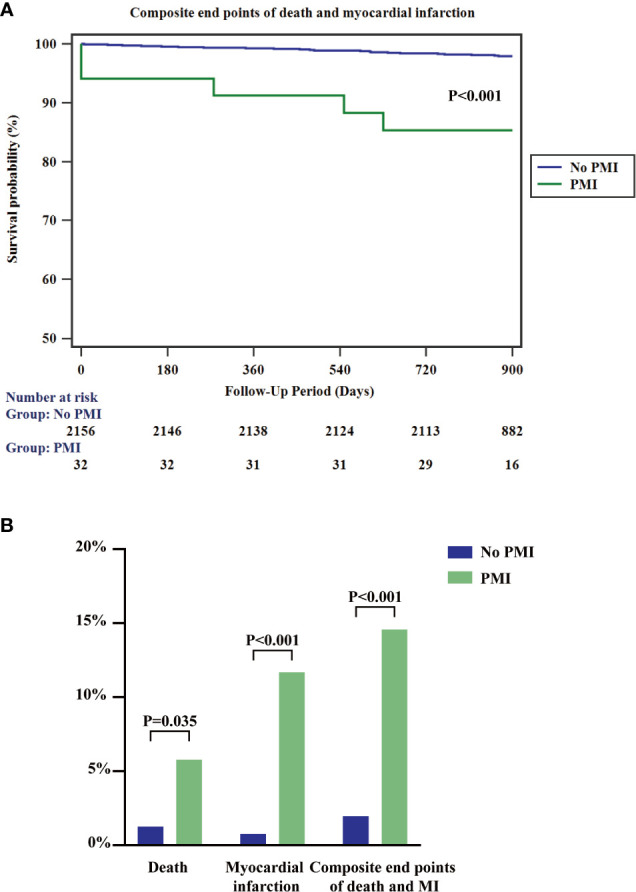
Kaplan-Meier survival curves for composite endpoints of all-cause mortality and myocardial infarction **(A)** and 2-year rates of the indicated events **(B)** according to the presence or absence of PMI. PMI, peri-procedural myocardial infarction; MI, myocardial infarction.

Lipid disorders commonly occur in patients with diabetes (2). Notably, lipoprotein abnormalities advance atherosclerosis and are a strong predictor of adverse cardiovascular outcomes. Lp(a) is an inheritable lipoprotein element, which is barely affected by dietary changes and environmental alterations (18). The ability of Lp(a) to engage in cardiovascular diseases mainly depends on its pro-atherosclerotic and pro-thrombotic effect (19). Lp(a) promotes the development of atherosclerosis by advancing foam cell formation, inducing smooth muscle cell proliferation, and activating monocyte chemoattractant (20). Moreover, Lp(a) could be a marker to predict the complexity and severity of the angiographic lesions. Prior studies reported that elevated Lp(a) was significantly associated with severe coronary conditions (21, 22). Besides, Lp(a) had a plasminogen-like function, which could bind to fibrin, inhibit fibrinolysis, and promote thrombosis (20). The increased frequency of PMI in our study provided additional evidence of the association of elevated Lp(a) and post-procedure elevated risk of thrombotic cardiac events. Moreover, the two-year composite outcomes of all-cause mortality and myocardial infarction significantly increased in the PMI group than the non-PMI group (**Figure 3**). Reducing Lp(a) level might provide a potential way to prevent PMI and subsequent adverse consequences.

Although broadly use of statin therapy (23) markedly decreases the risk of adverse cardiovascular events by targeting LDL (24), statin shows hardly any effect on lowering Lp(a) levels. Conversely, a recent meta-analysis revealed that Lp(a) levels escalated over 10% from baseline after statin treatment (25) and Lp(a) may represent part of the residual risk of the statin therapies (26). Hence, Lp(a) plays a critical role in contemporary lipid-lowering medication administration. In a recent BiomarCaRE (Biomarkers for Cardiovascular Risk Assessment in Europe) study (27), high Lp(a) levels robustly increased the risk of cardiac events in patients with DM, indicating that the Lp(a)-lowering might greatly improve the cardiac outcomes in diabetic patients. Our study further demonstrated that the low Lp(a) population had a reduced risk of procedure-related myocardial infarction and Lp(a) might be a potential evaluation tool for risk management in diabetic patients undergoing PCI.

The most promising Lp(a)-Lowering pharmacologic treatments are hepatocyte-directed antisense oligonucleotide (ASO) and proprotein convertase subtilisin/kexin type 9 (PCSK9) inhibitors. The latest randomized controlled trial of ASO therapy showed that Lp(a) achieved forcefully 35% to 72% reduction in a dose-dependent manner after 32 weeks (28). ODYSSEY OUTCOME (Evaluation of Cardiovascular Outcomes After an Acute Coronary Syndrome During Treatment With Alirocumab) randomized trial demonstrated that PCSK9 inhibitor alirocumab reduced Lp(a) by a median 23.5% after 4 months (29). Currently, lipoprotein apheresis is an efficient therapy for lowering Lp(a) levels (30). Recent evidence showed a reduction of 63% for Lp(a) had been achieved by apheresis and cardiovascular events markedly reduced about 94% after treatment (31). The combination of statin and Lp(a)-lowering therapy provides us an insight into future lipid treating therapy in patients with diabetes (32).

### Limitations

There are several limitations requiring careful consideration. First, this study was conducted in a single center, further multi-center validation is needed to confirm our findings. Second, the study was based on observational data, not on randomized design trials. Thus, the result of the analysis might not indicate final conclusions for the impact of Lp(a). Finally, although we adjusted the traditional risk factors, hidden unadjusted confounders may not be totally eliminated. Moreover, there was a lack of a family history record and genetic background that could allow us to further evaluate the inheritable Lp(a) influence.

### Conclusions

In conclusion, Lp(a) levels were independently associated with an elevated risk of PMI during PCI in male patients with DM. Lp(a) might be a significant indicator for PCI risk stratification and a potential target for PMI treatment. Further studies should be conducted to investigate the detailed role of Lp(a) in the pathology of PMI.

## Data Availability Statement

Request to the data of this study can be sent to the corresponding author, who will provide them to vetted and qualified applicants.

## Ethics Statement

The studies involving human participants were reviewed and approved by the Ethics Committee of Fuwai Hospital. The patients/participants provided their written informed consent to participate in this study.

## Author Contributions

YL: Conceptualization, methodology, investigation, formal analysis, and writing—original draft. WW: Data curation, writing—review and editing, and supervision. JS: Formal analysis, software, and validation. KZ: Project administration. BX: Resources. PL, CS, MY, JC: Investigation. Y-DT: Conceptualization, writing—review and editing, and supervision. All authors contributed to the article and approved the submitted version.

## Funding

This work was supported by the National Natural Science Foundation of China [81825003, 91957123]; the CAMS Innovation Fund for Medical Sciences [CIFMS 2016-I2M-1-009]; the Beijing Municipal Commission of Science and Technology (Z181100006318005); and the National Key Research and Development Program of China [2020YFC2004700].

## Conflict of Interest

The authors declare that the research was conducted in the absence of any commercial or financial relationships that could be construed as a potential conflict of interest.
