# Supplementary material for: Association Between Lipoprotein(a) and Peri-procedural Myocardial Infarction in Patients With Diabetes Mellitus Who Underwent Percutaneous Coronary Intervention
Source: Front Endocrinol (Lausanne). 2021 Feb 3;11:603922. doi: 10.3389/fendo.2020.603922 (PMC7888338; doi:10.3389/fendo.2020.603922)
Supplement: Supplementary file 1 [file Table_1.docx]

**Supplementary Table S1.** Baseline characteristics and procedural characteristics of patients with or without available lipoprotein(a) data.

|  | Lp(a) not available (N=1052) | Lp(a) available (N=2190) | P Value |
| --- | --- | --- | --- |
| Age | 59.10±9.73 | 59.31±9.76 | 0.573 |
| Male | 776 (73.8) | 1657 (75.7) | 0.242 |
| BMI | 26.17±3.22 | 26.26±3.16 | 0.411 |
| Current smoking | 575 (54.7) | 1212 (55.3) | 0.714 |
| Hypertension | 732 (69.6) | 1537 (70.2) | 0.727 |
| Hyperlipidemia | 776 (73.8) | 1633 (74.6) | 0.625 |
| Previous CABG surgery | 53 (5.0) | 105 (4.8) | 0.763 |
| Peripheral vascular disease | 30 (2.9) | 99 (4.5) | 0.023 |
| Previous cerebrovascular disease | 126 (12.0) | 303 (13.8) | 0.144 |
| Total cholesterol, mmol/L | 4.90±2.06 | 4.15±1.08 | 0.332 |
| HDL, mmol/L | 0.96±0.38 | 1.01±0.28 | 0.752 |
| LDL, mmol/L | 3.14±2.03 | 2.45±0.90 | 0.278 |
| HbA1c | 7.84±1.32 | 7.80±1.43 | 0.862 |
| Creatinine, μmoI/L | 75.58±17.90 | 75.15±16.78 | 0.504 |
| LVEF | 62.17±7.60 | 62.63±7.53 | 0.106 |
| Multivessel disease | 853 (81.1) | 1814 (82.8) | 0.223 |
| Left main disease | 69 (6.6) | 169 (7.7) | 0.237 |
| Bifurcation lesion | 398 (37.8) | 900 (41.1) | 0.076 |
| Total occlusion | 84 (8.0) | 184 (8.4) | 0.686 |
| Target vessel diameter, mm | 3.15±1.78 | 3.09±1.04 | 0.245 |
| Percent stenosis | 89.41±8.14 | 88.80±8.21 | 0.047 |
| Total treated lesion length, mm | 28.48±18.26 | 30.08±18.72 | 0.021 |
| Moderate to severe calcification | 186 (17.7) | 389 (17.8) | 0.954 |
| Moderate to severe angulation | 95 (9.0) | 193 (8.8) | 0.838 |
| SYNTAX score | 12.43±8.44 | 12.21±8.34 | 0.487 |
| Number of stents | 1.84±1.12 | 1.89±1.18 | 0.218 |
| Procedural duration, min | 36.60±29.79 | 36.03±31.40 | 0.618 |

^a^. Variables are shown as median±SD or n (%). Lp(a), lipoprotein(a); CABG, coronary artery bypass graft; BMI, body mass index; LVEF, left ventricular ejection function; HDL, high-density lipoprotein; LDL, low-density lipoprotein; HbA1c, haemoglobin A1c.
